# Development and validation of a predictive model for acute exacerbation in chronic obstructive pulmonary disease patients with comorbid insomnia

**DOI:** 10.3389/fmed.2025.1511874

**Published:** 2025-03-21

**Authors:** Qianqian Gao, Hongbin Zhu

**Affiliations:** ^1^Chaohu Hospital Affiliated with Anhui Medical University, Chaohu, China; ^2^Department of Respiratory and Critical Care Medicine, Chaohu Hospital Affiliated with Anhui Medical University, Chaohu, China

**Keywords:** COPD – Chronic obstructive pulmonary disease, Insomnia, Comorbidity, Nomogram, Prediction model

## Abstract

**Aim:**

To develop and validate a risk prediction model for estimating the likelihood of insomnia in patients with acute exacerbations of chronic obstructive pulmonary disease (AECOPD).

**Methods:**

This prospective study enrolled 253 patients with AECOPD treated at the Department of Respiratory and Critical Care Medicine, Chaohu Hospital Affiliated with Anhui Medical University, between September 2022 and April 2024. Patients were randomly assigned to a training set and a testing set in a 7:3 ratio. Least Absolute Shrinkage and Selection Operator (LASSO) regression analysis was conducted in the training set to identify factors associated with insomnia in patients with AECOPD. A nomogram was constructed based on four identified variables to visualize the prediction model. Model validation involved the Hosmer-Lemeshow test, and its performance was assessed through receiver operating characteristic (ROC) curves, calibration curves, and decision curve analysis (DCA). Model interpretability was further enhanced using SHapley Additive exPlanations (SHAP).

**Results:**

PSQI grade, marital status (widowed), white blood cell (WBC) count, and eosinophil percentage (EOS%) were identified as significant predictors of insomnia in patients with AECOPD. The nomogram based on these predictors exhibited excellent predictive performance, with areas under the ROC curve (AUCs) of 0.987 and 0.933 for the training and testing sets, respectively. The calibration curves and Hosmer-Lemeshow test demonstrated strong agreement between predicted and observed outcomes, while DCA confirmed the model’s superior clinical utility.

**Conclusion:**

This study established a risk prediction model based on four variables to estimate the probability of insomnia in patients with AECOPD. The model exhibited excellent predictive accuracy and clinical applicability, offering valuable guidance for early identification and management of insomnia in this population.

## 1 Introduction

Chronic obstructive pulmonary disease (COPD) is a prevalent lung disorder characterized by airflow limitation and breathing difficulties. It has emerged as the third leading cause of death globally (1). In 2019, the prevalence of COPD among individuals aged 30–79 ranged from 7.6 to 10.6% (2), with the disease responsible for over 3 million deaths that year (3). The Global Burden of Disease Study reported that in 2022, COPD was the second leading cause of disability-adjusted life years and mortality worldwide, surpassed only by cardiovascular diseases. This significant health burden is expected to persist through 2050 (4).

The primary symptoms of COPD are dyspnea and chronic cough with sputum production, while insomnia is also a common comorbidity (5). Insomnia activates inflammatory pathways, triggering the release of pro-inflammatory cytokines such as TNF-*α* and IL-6 (6). Additionally, it contributes to oxidative stress by promoting free radical production and suppressing antioxidant mechanisms, leading to redox imbalance (7). These processes intensify systemic inflammation in COPD patients, further aggravating respiratory symptoms and diminishing sleep quality. Furthermore, airflow limitation and lung hyperinflation associated with COPD exacerbate hypoventilation during sleep, resulting in hypoxemia and hypercapnia, which increase the frequency of awakenings (8). Chronic hypoxia activates the sympathetic nervous system, causing an elevated heart rate, increased blood pressure, and psychological disturbances such as anxiety and worry, all of which further contribute to insomnia (9, 10). Insomnia in COPD patients is also linked to mental health disorders such as depression and anxiety. Additionally, common treatments for COPD—including corticosteroids (10), quinolone antibiotics (11), and the use of ventilatory support (12)—may adversely affect sleep quality.

Patients with COPD and comorbid insomnia frequently exhibit reduced sleep efficiency, poor sleep quality, and an increased tendency toward fatigue, attention deficits, and excessive daytime sleepiness (13). Insomnia may also contribute to a range of adverse outcomes, including heightened risks of anxiety, depression, and cognitive impairment, along with increased reliance on sedatives and the potential development of cardiovascular complications (14). Additionally, chronic hypoxia and sleep deprivation can intensify systemic inflammation, drive disease progression, and elevate the risk of acute exacerbations of COPD (AECOPD) and associated mortality (15).

Previous studies have primarily focused on exploring the associations between AECOPD and common hematological indicators, such as routine blood tests and liver and kidney function, while fewer have investigated patients’ psychological status or treatment-related factors (16, 17). In recent years, the application of emerging technologies like machine learning has underscored the significant role of psychological status and lifestyle factors in predicting insomnia (18). Additionally, by integrating various clinical and non-clinical features, some studies have successfully developed risk prediction models for acute exacerbations of AECOPD, offering new perspectives on understanding patients’ overall health (19). Building on previous research regarding insomnia in patients with AECOPD, this study integrates patients’ clinical characteristics and treatment methods—particularly the use of corticosteroids, quinolone antibiotics, and ventilatory support—to further explore the associated risk factors. A nomogram-based risk prediction model for insomnia was developed, providing clinicians with a quantitative and visually intuitive tool for the early identification of high-risk patients and the design of personalized interventions. This approach not only enhances treatment strategies but also reduces the disease burden, ultimately improving the patients’ overall quality of life.

## 2 Materials and methods

### 2.1 Study design and participants

We collected data from 253 patients experiencing AECOPD who were treated at Chaohu Hospital Affiliated with Anhui Medical University between September 2022 and April 2024.

Inclusion criteria included: (1) Diagnosis of COPD based on the *Global Initiative for Chronic Obstructive Lung Disease 2023 Report: GOLD Executive Summary* (20): Patients present with dyspnea, chronic cough, sputum production, a history of recurrent lower respiratory tract infections, and exposure to risk factors for the disease. Pulmonary function tests performed during either a previous or current hospitalization reveal FEV_1_/FVC < 70% following bronchodilator administration, with other diagnoses excluded, confirming the COPD diagnosis. (2) Hospitalization due to acute exacerbation: Symptoms have worsened rapidly, requiring modifications to the treatment plan.

Exclusion criteria included: (1) those with a diagnosed history of mental illness or severe organ failure. (2) Individuals who had used antidepressants, sedatives, or long-term sleep medications in the past month. (3) Patients with impaired consciousness or language function. (4) Individuals unable to complete the survey for any reason. (5) Patients with other conditions in the past month that significantly affected sleep quality, such as trauma or fractures. (6) Patients with incomplete clinical or pathological data.

This study was conducted in accordance with the Declaration of Helsinki and received approval from the Ethics Committee of Chaohu Hospital Affiliated to Anhui Medical University (Approval Number: KYXM-202209-009). In addition, informed consent has been obtained from all participating individuals.

### 2.2 Clinical baseline data

Clinical and pathological data were collected from patients, including age, gender, smoking history, marital status (widowed or not), education level, body mass index (BMI), comorbidities, number of hospitalizations in the past year, mMRC grade, and CAT score. The Pittsburgh Sleep Quality Index (PSQI) and Insomnia Severity Index (ISI) were used to assess sleep quality over the past month. Laboratory tests performed within 24 h of admission included routine blood tests, coagulation profile, liver and kidney function, and trace elements. Additionally, detailed information regarding medications, ventilator use, and any resuscitation measures taken during the hospitalization was recorded.

### 2.3 Assessment of sleep status

The Insomnia Severity Index (ISI) is used to assess sleep patterns and the impact of insomnia over a two-week period. The scale consists of seven questions, each rated from 0 to 4 (0 = none, 4 = very much or severe). A total score of ≤7 indicates no clinically significant insomnia, while a score > 7 suggests the presence of insomnia.

The Pittsburgh Sleep Quality Index (PSQI) is a scale used to evaluate nighttime sleep quality and sleep behaviors over the past month. It includes 19 items, with a total score ranging from 0 to 21. A higher score reflects poorer sleep quality: a score of ≤5 indicates excellent sleep quality, 6–10 indicates good sleep quality, 11–15 indicates fair sleep quality, and 16–21 indicates very poor sleep quality.

All patients completed the questionnaires on the first day of hospitalization before receiving any treatment. Reassessments were performed on the third day of treatment and on the final day of hospitalization. For the final data analysis, the assessment score on the last day of treatment was used.

### 2.4 Statistical analysis

Statistical analyses were conducted using SPSS version 25.0 and R software (version 4.4.1). Continuous variables with a normal distribution and homogeneity of variance are expressed as mean ± standard deviation (*x-* ± *s*) and were analyzed using independent sample t-tests. Data that did not follow a normal distribution or exhibited heterogeneous variances are presented as median (interquartile range) [*M* (P25–P75)] and were analyzed using non-parametric tests. Categorical variables are presented as frequency [*n* (%)] and were compared using the chi-square test. Patients were randomly divided into training and testing sets in a 7:3 ratio, and independent risk factors were selected using the Least Absolute Shrinkage and Selection Operator (LASSO) regression. The goodness-of-fit and validity of the risk model were assessed using the Hosmer-Lemeshow test. The nomogram model was developed using the “rms” package. The receiver operating characteristic (ROC) curve was plotted to calculate the area under the curve (AUC) for the predictive model, and its discrimination, calibration, and clinical utility were evaluated using the calibration curve and decision curve analysis (DCA). The model was interpreted using SHAP. All statistical analyses were two-tailed, and statistical significance was set at *p* < 0.05.

## 3 Results

### 3.1 Basic clinical and pathological information of all included patients

This study collected data from 253 patients with AECOPD, of which 19 were excluded due to incomplete medical records, leaving 234 patients for analysis. The patients were grouped based on their ISI scores, with those scoring >7 classified into the insomnia group (*n* = 130) and those scoring ≤7 as the control group (*n* = 104). The overall prevalence of insomnia in patients with AECOPD was 55.6%. A comparison of the baseline clinical and pathological data revealed significant differences between the insomnia and control groups in terms of education level, PSQI scores, CAT scores, mMRC grades, and the number of hospitalizations in the past year (*p* < 0.05), as shown in Table 1.

**Table 1:** Basic clinical and pathological information of all included patients.

Variables	Insomnia group (*n* = 130)	Control group (*n* = 104)	*Z*/*χ*^2^	*P*-value
Gender			3.248	0.071
Male	99 (76.2%)	89 (85.6%)		
Female	31 (23.8%)	15 (14.4%)		
Age (years)	75 (71 ~ 81)	77 (71 ~ 80)	−0.456	0.648
BMI (kg/m^2^)	20.74 (18.90 ~ 23.20)	21 (19.11 ~ 23.20)	−0.538	0.590
Smoking duration /years	30 (0 ~ 50)	40 (20 ~ 50)	−1.573	0.116
Smoking cessation			2.031	0.154
Yes	95 (73.1%)	67 (64.4%)		
No	35 (26.9%)	37 (35.6%)		
Widowed			1.211	0.271
Yes	45 (34.6%)	29 (27.9%)		
No	85 (65.4%)	75 (72.1%)		
Education level			5.630	0.018
Primary school or below	103 (79.2%)	68 (65.4%)		
Above primary school	27 (20.8%)	36 (34.6%)		
Comorbidities			0.672	0.412
Yes	72 (55.4%)	52 (50.0%)		
No	58 (44.6%)	52 (50.0%)		
Emergency hospitalization			0.175	0.676
Yes	12 (9.2%)	8 (7.7%)		
No	118 (90.8%)	96 (92.3%)		
Use of ventilator			0.101	0.751
Yes	22 (16.9%)	16 (15.4%)		
No	108 (83.1%)	88 (84.6%)		
Use of quinolone drugs			1.408	0.235
Yes	31 (23.8%)	32 (30.8%)		
No	99 (76.2%)	72 (69.2%)		
Intravenous corticosteroids			3.601	0.058
Yes	94 (72.3%)	63 (60.6%)		
No	36 (27.7%)	41 (39.4%)		
PSQI classification			−11.721	<0.001
Excellent	1 (0.8%)	35 (33.7%)		
Good	15 (11.5%)	60 (57.7%)		
Fair	88 (67.7%)	9 (8.7%)		
Very poor	26 (20.0%)	0 (0.0%)		
CAT score			−6.303	<0.001
Mild	5 (3.8%)	8 (7.7%)		
Moderate	26 (20.0%)	59 (56.7%)		
Severe	68 (52.3%)	32 (30.8%)		
Very severe	31 (23.8%)	5 (4.8%)		
mMRC grade			−2.995	0.003
Grade 0	0 (0.0%)	1 (1.0%)		
Grade 1	8 (6.2%)	11 (10.6%)		
Grade 2	27 (20.8%)	28 (26.9%)		
Grade 3	55 (42.3%)	50 (48.1%)		
Grade 4	40 (30.8%)	14 (13.5%)		
Exacerbation frequency in the past year	1 (1 ~ 2)	1 (1 ~ 2)	−2.721	0.007
Hospitalization days	8 (6 ~ 10)	7 (6 ~ 9)	−1.910	0.056

### 3.2 Clinical and pathological data of the training and testing sets

All enrolled patients were randomly assigned to a training set and a testing set in a 7:3 ratio, and their clinical data were subjected to statistical analysis. The training set comprised 164 AECOPD patients, with 91 (55.5%) having comorbid insomnia. The testing set included 70 AECOPD patients, 39 (55.7%) of whom had comorbid insomnia. No significant differences were observed between the two groups regarding insomnia prevalence, laboratory results, or treatment measures (*p* > 0.05). Detailed data are provided in Table 2.

**Table 2:** Clinical and pathological data of the training and testing sets.

Variables	Training set (*n* = 164)	Testing set (*n* = 70)	*Z*/*χ*^2^/*t*	*P*-value
Comorbid insomnia			0.001	0.975
Yes	91 (55.50%)	39 (55.70%)		
No	73 (44.50%)	31 (44.30%)		
Gender			0.075	0.785
Male	131 (79.90%)	57 (81.40%)		
Female	33 (20.10%)	13 (18.60%)		
Age (years)	75.00 (71.00, 81.00)	76.00 (70.00, 80.00)	−0.303	0.762
BMI (kg/m^2^)	20.36 (18.90, 23.21)	21.20 (19.14, 23.60)	−0.913	0.361
Smoking duration /years	35.00 (1.25, 50.00)	40.00 (0, 50.00)	−0.299	0.765
Smoking cessation			0.020	0.886
Yes	114 (69.50%)	48 (68.60%)		
No	50 (30.50%)	22 (31.40%)		
Widowed			0.327	0.567
Yes	114 (69.50%)	46 (65.70%)		
No	50 (30.50%)	24 (34.30%)		
Education level			0.002	0.961
Primary school or below	120 (73.20%)	51 (72.90%)		
Above primary school	44 (26.80%)	19 (27.10%)		
Comorbidities			1.248	0.264
Yes	83 (50.60%)	41 (58.60%)		
No	81 (49.40%)	29 (41.40%)		
Emergency hospitalization			0.000	0.993
Yes	14 (8.50%)	6 (8.60%)		
No	150 (91.50%)	64 (91.40%)		
Use of ventilator			0.399	0.527
Yes	25 (15.20%)	13 (18.60%)		
No	139 (84.80%)	57 (81.40%)		
Use of quinolone drugs			0.002	0.961
Yes	44 (26.80%)	19 (27.10%)		
No	120 (73.20%)	51 (72.90%)		
Intravenous corticosteroids			0.357	0.550
Yes	112 (68.30%)	45 (64.30%)		
No	52 (31.70%)	25 (35.70%)		
PSQI classification			−0.232	0.816
Excellent	23 (14.00%)	13 (18.60%)		
Good	58 (35.40%)	17 (24.30%)		
Fair	64 (39.00%)	33 (47.10%)		
Very poor	19 (11.60%)	7 (10.00%)		
CAT score			0.679	0.497
Mild	10 (6.10%)	3 (4.30%)		
Moderate	57 (34.80%)	28 (40.00%)		
Severe	69 (42.10%)	31 (44.30%)		
Very severe	28 (17.10%)	8 (11.40%)		
mMRC grade			−0.335	0.737
Grade 0	0 (0%)	1 (1.40%)		
Grade 1	16 (9.80%)	3 (4.30%)		
Grade 2	37 (22.60%)	18 (25.70%)		
Grade 3	70 (42.70%)	35 (50.00%)		
Grade 4	41 (25.00%)	13 (18.60%)		
Exacerbation frequency in the past year	1(1, 2)	1 (1, 2)	−1.304	0.192
Hospitalization days	8 (6, 9)	7 (6, 10)	−0.358	0.720
WBC (×10^9^/L)	7.18 (5.28, 9.75)	6.23 (4.84, 8.71)	−1.302	0.193
Neu (%)	75.15 (64.53, 83.35)	72.85 (61.70, 81.33)	−1.345	0.179
EOS (%)	0.30 (0.00, 2.08)	0.50 (0.00, 2.53)	−0.560	0.575
NEU (×10^9^/L)	5.27 (3.31, 7.98)	4.34 (3.23, 6.74)	−1.570	0.116
E (×10^9^/L)	0.02 (0.00, 0.13)	0.04 (0.00, 0.14)	−0.413	0.679
RBC (×10^12^/L)	4.13 ± 0.60	4.16 ± 0.56	0.358	0.721
Hb (g/L)	124.38 ± 18.08	127.53 ± 18.01	1.219	0.224
PLT (×10^9^/L)	179.50 (139.25, 233.00)	168.00 (133.50, 237.25)	−0.884	0.377
PT (s)	10.80 (10.40, 11.70)	10.90 (10.40, 11.53)	−0.024	0.981
PT% (%)	90.44 ± 12.12	90.24 ± 11.10	−0.118	0.906
APTT (s)	28.60 (26.80, 30.48)	29.00 (27.18, 30.50)	−0.950	0.342
FIB (g/L)	4.17 (3.13, 5.66)	3.68 (2.94, 5.04)	−1.591	0.112
TT (s)	17.31 ± 1.44	17.81 ± 2.76	1.799	0.073
D-dimer (μg/mL)	0.51 (0.32, 0.94)	0.48(0.33, 0.99)	−0.296	0.767
ALT (U/L)	17.00 (12.00, 25.00)	16.50 (12.75, 26.50)	−0.119	0.905
AST (U/L)	22.00 (17.00, 29.00)	21.00 (18.00, 30.00)	−0.001	0.999
TBIL (μmol/L)	12.95 (9.00, 17.00)	11.00 (8.00, 14.25)	−1.718	0.086
TP (g/L)	66.11 ± 6.60	65.03 ± 7.81	−1.085	0.279
ALB (g/L)	38.60 ± 4.42	38.53 ± 4.22	−0.098	0.922
BUN (mmol/L)	6.50 (5.40, 7.90)	6.10 (4.98, 7.70)	−1.237	0.216
Scr (μmol/L)	67.00 (56.25, 83.00)	69.50 (58.00, 88.25)	−1.212	0.226
UA (μmol/L)	290.00 (235.00, 360.75)	292.50 (226.75, 369.00)	−0.014	0.989
Cu (μmol/L)	20.10 (16.73, 24.30)	20.00 (17.28, 23.73)	−0.498	0.619
Fe (mmol/L)	8.57 (7.50, 9.81)	8.85 (7.72, 9.45)	−0.874	0.382
Mg (mmol/L)	1.49 (1.32, 1.76)	1.49 (1.34, 1.71)	−0.418	0.676
Zn (μmol/L)	111.55 ± 20.10	116.12 ± 24.39	1.493	0.137
Ca (mmol/L)	1.63 (1.43, 1.98)	1.70 (1.46, 1.97)	−0.712	0.477
Pb (μg/L)	23.00 (12.00, 34.00)	24.00 (10.75, 37.00)	−0.246	0.806

### 3.3 Selection of predictive factors

**Figure 1:**
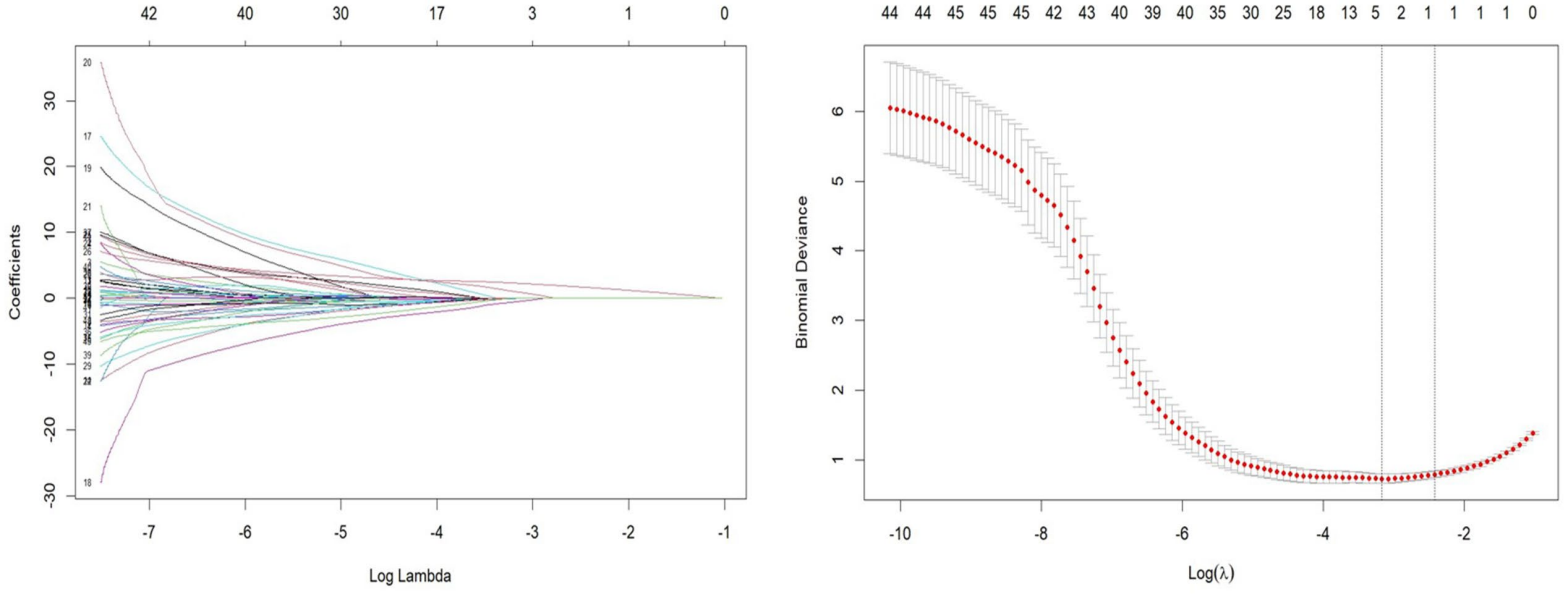
Least absolute shrinkage and selection operator (LASSO) regression path and cross-validation results.

Patients were divided into a testing set and a training set using the R function “createDataPartition,” resulting in a testing set of 164 patients and a training set of 70 patients. The LASSO regression model was applied to the 164 patients in the training set to select features with non-zero coefficients. As the penalty coefficient varied, the number of variables in the model gradually decreased. Based on 20-fold cross-validation, the model exhibited excellent performance when the optimal *λ* value was 0.042 (Log *λ* = −5.322), as shown in Figure 1. At this stage, we identified four predictive factors for the development of the model: PSQI grading, marital status (whether widowed), white blood cell (WBC), and eosinophil percentage (EOS%).

### 3.4 Risk prediction model

**Figure 2:**
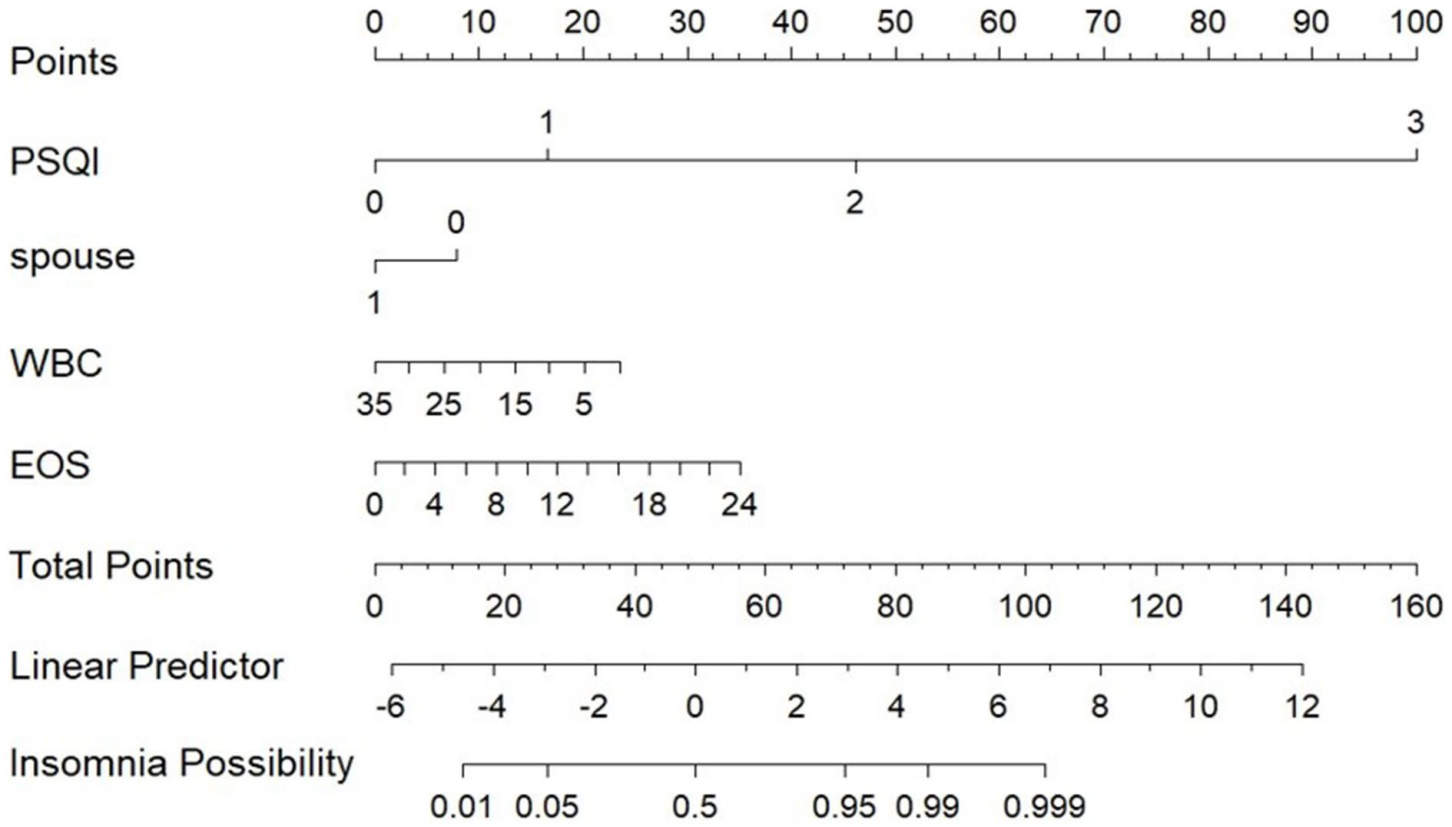
Predictive model of insomnia in patients with AECOPD.

Using LASSO regression analysis, this study developed a risk prediction model for insomnia in patients with AECOPD, which was visually represented with a nomogram (see Figure 2). The nomogram includes four predictive variables: PSQI grade, marital status, WBC, and EOS%. Each predictive factor’s score is mapped to the scoring axis at the top of the nomogram. By summing the scores of all variables, a total score is calculated. This total score is then matched to its corresponding position on the total score axis and extended vertically to the probability axis, providing the likelihood of insomnia in patients with AECOPD. For instance, a total score of 60 corresponds to an insomnia probability of over 50%, while a score of 80 indicates a probability exceeding 95%.

### 3.5 Model validation and evaluation

**Figure 3:**
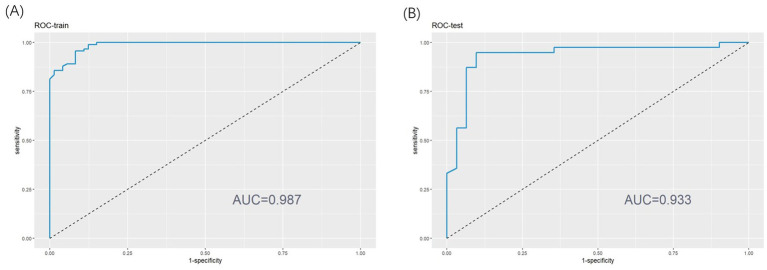
**(A)** Receiver operating characteristic (ROC) curve of training set. **(B)** ROC curve of test set.

This study utilized ROC analysis, calibration curves, and decision curve analysis (DCA) to assess the predictive efficiency of insomnia probability in patients with AECOPD. The results demonstrated that the nomogram exhibited excellent predictive performance. For the training set, the area under the curve (AUC) was 0.987 (95% CI, 0.976–0.998) (as shown in Figure 3A), while the AUC for the testing set was 0.933 (95% CI, 0.8671–1.000) (as shown in Figure 3B). These AUC values indicate the strong diagnostic capability of the nomogram in both datasets.

**Figure 4:**
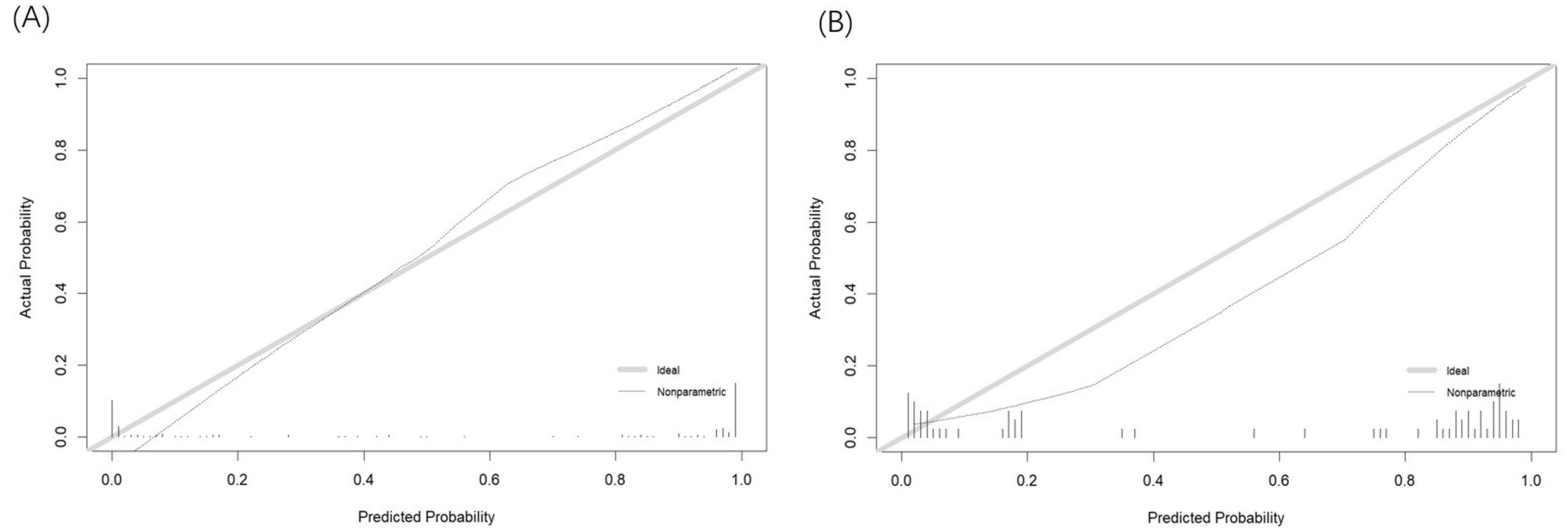
**(A)** Calibration curve of training set. **(B)** Calibration curve of test set.

The Hosmer-Lemeshow test confirmed that the model is consistent with the observed data (*p* > 0.05). In the calibration curve, the *X*-axis represents the predicted probability of insomnia in patients with AECOPD, while the *Y*-axis shows the actual observed probability. The diagonal line represents the ideal model (where predicted values equal observed values). The closer the calibration curve is to the diagonal, the more accurate the model’s predictions. As illustrated in Figures 4A,B, the calibration curve closely aligns with the ideal curve, indicating that the nomogram-based risk prediction model demonstrates strong predictive performance and high reliability.

**Figure 5:**
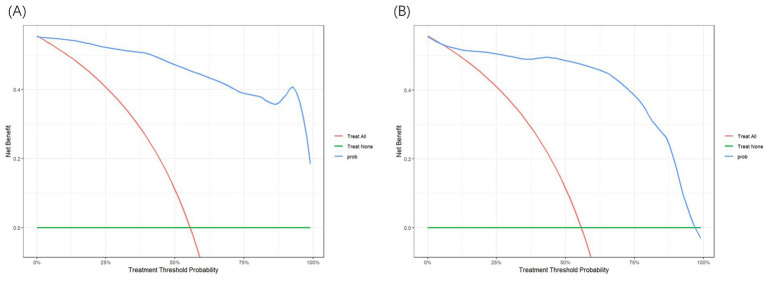
**(A)** Decision curve analysis (DCA) curve of training set. **(B)** DCA curve of test set.

This study also evaluated the clinical effectiveness of the model using Decision Curve Analysis (DCA). Figures 5A,B display the DCA curves for the training and validation sets, respectively. In these figures, the *X*-axis represents the threshold probability, and the *Y*-axis represents the net benefit. The green reference line shows the net benefit without any intervention, while the red curve represents the net benefit when all patients receive intervention. In the threshold probability range of 0.1–0.9, the blue curve consistently lies above the reference line, indicating that the prediction model delivers a significant net benefit across most threshold probabilities. This finding supports the model’s effectiveness and clinical applicability.

### 3.6 SHAP interpretation of the model

**Figure 6:**
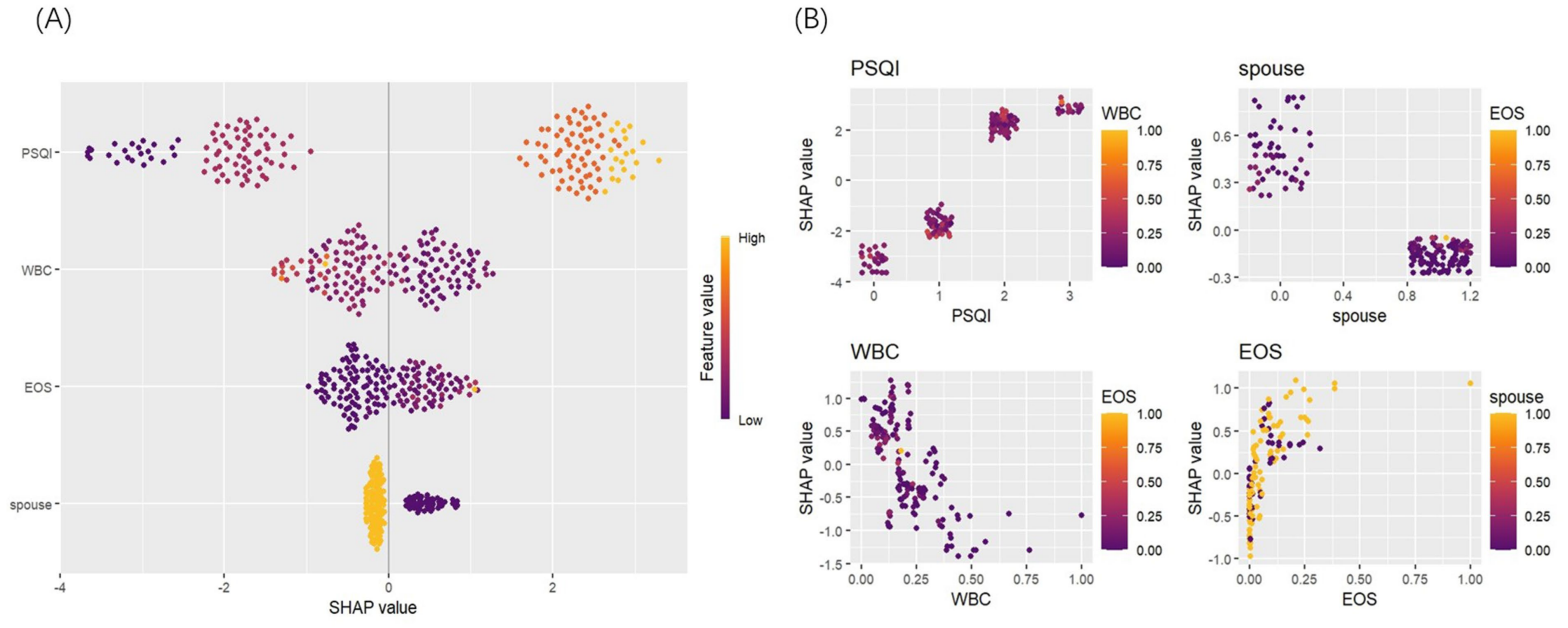
SHAP: **(A)** feature importance plot. **(B)** Partial dependence plot.

Accurately identifying the key factors influencing insomnia is crucial for developing an effective prediction model. By comparing feature importance using statistical methods, the model’s predictive accuracy and interpretability can be optimized (21). In this study, SHAP was employed for a visual analysis of the importance and dependencies of the selected predictive factors. The results revealed that four variables—PSQI, marital status, WBC, and EOS%—significantly impacted the prediction of insomnia in patients with AECOPD. PSQI score emerged as the most important predictor. Its dependence chart showed a strong correlation between higher PSQI scores and an increased risk of insomnia, with substantial variability. WBC levels also notably influenced the risk of insomnia, with higher WBC levels associated with a greater likelihood of insomnia. Additionally, the variable importance chart highlighted that marital status (being widowed) played a significant role in the model, while WBC and EOS% also contributed to predicting insomnia. Specifically, higher PSQI scores and elevated WBC levels were positively correlated with an increased risk of insomnia, while changes in EOS% were also closely linked to the occurrence of insomnia (Figure 6).

**Figure 7:**
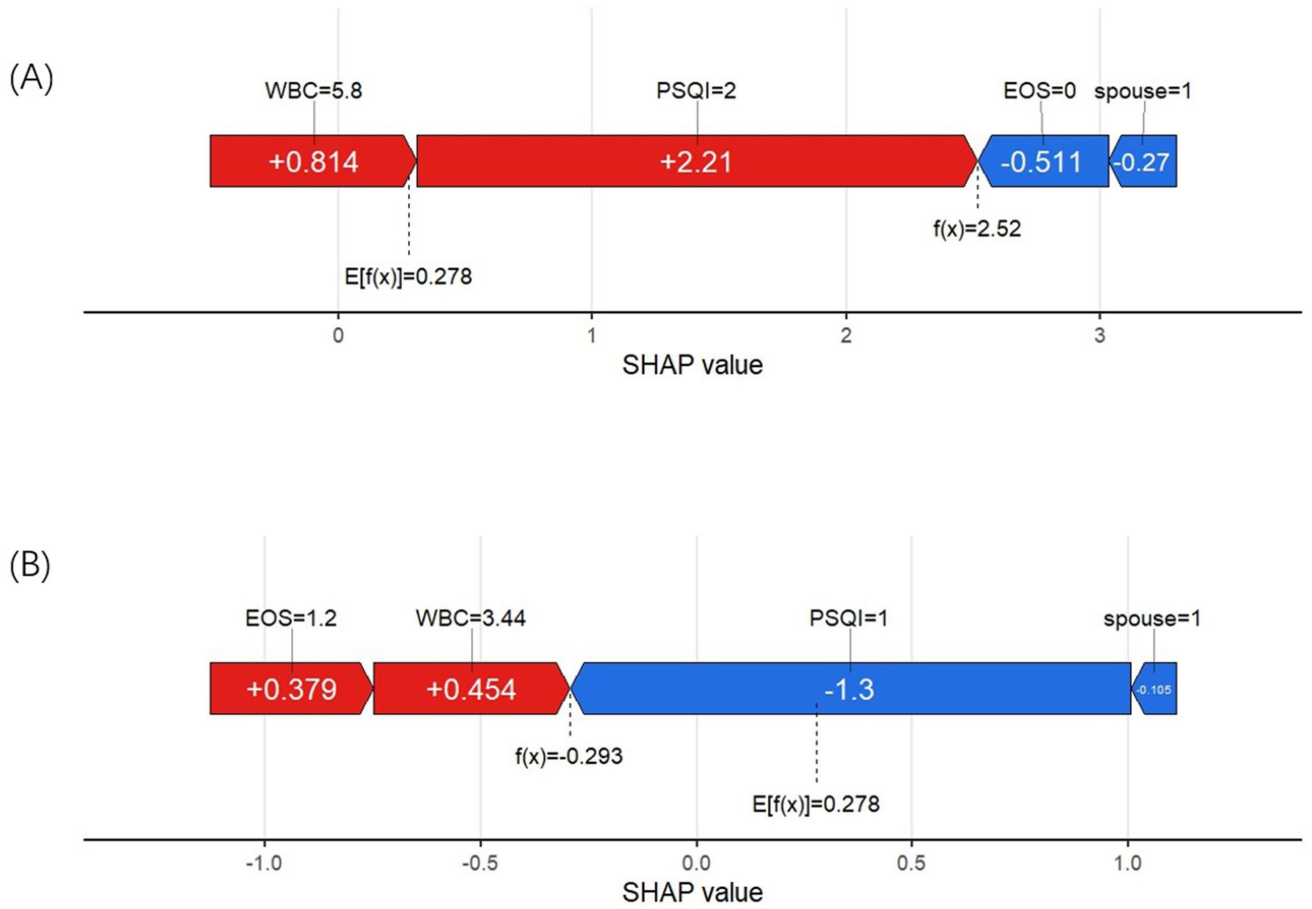
**(A)** SHAP force plot of a positive result cases. **(B)** SHAP force plot of a negative result case.

We used two examples to illustrate how each variable influences the prediction outcome for each sample. In Figure 7A, the model predicts a positive result for an AECOPD patient who actually developed insomnia. The longest red segment in the figure corresponds to the PSQI grade (Level 2), indicating that PSQI grade has the greatest positive contribution to this patient’s insomnia outcome. The second most significant positive influence on the outcome is the WBC level (5.8 * 10^9/L). In Figure 7B, the model predicts a negative result for a patient who did not develop insomnia. The variables with positive impacts include WBC (3.44 * 10^9/L) and EOS% (1.2%). The most influential variables on the outcome are PSQI grade (Level 1) and marital status (not widowed).

## 4 Discussion

With the increasing trend of aging, COPD has become more prevalent worldwide, presenting a significant public health challenge. It predominantly affects low- and middle-income countries, with a particularly high prevalence among the elderly population (22). COPD is not confined to the lungs; it is a systemic disease that often impacts the cardiovascular, endocrine, and hematologic systems. Moreover, COPD patients are frequently confronted with psychological disorders such as anxiety, depression, and insomnia (23, 24). Insomnia is widespread among COPD patients and is strongly linked to an increased risk of acute exacerbations (15). Comprehensive assessments and effective interventions for COPD patients with insomnia are crucial in reducing the likelihood of AECOPD and alleviating the associated disease burden.

As such, developing a reliable tool to identify and predict the risk of insomnia in these patients is essential. By using personalized predictive scoring, the model can pinpoint key factors influencing insomnia and provide a quantified risk assessment. Based on these results, clinicians can create more targeted treatment and intervention plans, such as offering psychological counseling to high-risk patients, implementing strategies to improve sleep quality, and closely monitoring systemic inflammation and other relevant indicators. This approach not only improves overall patient care but also enhances COPD treatment outcomes, reduces complications linked to insomnia, and ultimately improves patient prognosis and quality of life.

This study included 234 patients, and the results revealed that the insomnia group had significantly poorer sleep quality scores compared to the control group (*p* < 0.05). Significant differences were observed between the two groups in CAT scores, mMRC grades, and the number of hospitalizations over the past year (*p* < 0.05), with the insomnia group exhibiting more severe COPD symptoms, higher scores, and higher grades. LASSO regression identified four variables significantly associated with insomnia in patients with AECOPD: PSQI grade, marital status (widowed), WBC levels, and EOS%. Using the risk prediction model, the SHAP method was applied to interpret the results. The model was validated with ROC curves, calibration curves, and DCA. The validation results demonstrated strong discrimination and calibration abilities, highlighting the model’s high clinical applicability and its potential as an effective tool for identifying high-risk patients.

This study identified the PSQI score as a significant risk factor for insomnia in patients with AECOPD. The PSQI assesses various aspects of sleep, such as sleep latency, sleep efficiency, and the frequency of nocturnal awakenings, providing a comprehensive measure of sleep quality. Patients with AECOPD often experience nocturnal hypoxemia, which activates the sympathetic nervous system, leading to increased heart rate, elevated blood pressure, and disrupted sleep, all of which contribute to reduced overall sleep efficiency (9, 10). Additionally, AECOPD patients frequently face issues such as inadequate ventilation and ventilation-perfusion mismatch, especially when lying in a supine position. Airflow obstruction can cause lung hyperinflation, further increasing respiratory strain. This worsens oxygen exchange, resulting in decreased blood oxygen saturation and increased carbon dioxide retention. Elevated carbon dioxide levels may lead to hypercapnia, exacerbating dyspnea. These pathophysiological changes make patients more susceptible to sleep disturbances and frequent awakenings during the night, severely impairing sleep quality (12, 25). Furthermore, the decline in subjective sleep quality can intensify anxiety and depressive symptoms, creating a vicious cycle that perpetuates and worsens insomnia (26). Thus, the PSQI score serves not only as a crucial predictor of insomnia in AECOPD patients but also indicates that those with higher PSQI scores are more likely to experience insomnia symptoms.

The results of this study indicate that widowhood is a significant risk factor for insomnia in patients with AECOPD. In China, men typically have higher smoking rates, and smoking is one of the main risk factors for COPD, closely linked to the onset of the disease. On the other hand, women often bear a heavier household burden and are frequently exposed to cooking fumes, which may also increase the risk of developing AECOPD (27). Widowhood is often accompanied by psychological stress and emotional trauma, making individuals more susceptible to anxiety, depression, and other negative emotions. These emotional struggles can, in turn, exacerbate insomnia (13). Moreover, widowed individuals often face greater financial pressures and poorer living or working conditions, which may adversely affect the management of AECOPD and significantly increase the risk of insomnia.

The study results suggest that patients with AECOPD and higher WBC levels are more likely to experience insomnia symptoms than those with lower WBC levels. As an easily accessible marker, white blood cell count (WBC) may play a role in the development of insomnia in AECOPD patients through various mechanisms, including inflammation, immune modulation, and sleep disturbances. AECOPD patients are typically in a state of chronic inflammation, and insomnia is closely linked to the release of pro-inflammatory cytokines. Elevated inflammation levels may worsen sleep disturbances by activating the hypothalamic–pituitary–adrenal (HPA) axis and the sympathetic nervous system (28). Furthermore, increased endotoxin levels in the blood of AECOPD patients may stimulate the release of inflammatory markers such as interleukin-6 (IL-6) and tumor necrosis factor-alpha (TNF-*α*) (29, 30), amplifying the inflammatory response and further disrupting sleep quality. Oxidative stress is another critical mechanism at play. Persistent oxidative stress and inflammation can disrupt the regulation of the nervous system, thereby increasing the risk of insomnia (7, 31). This interaction between inflammation and insomnia may create a vicious cycle that negatively impacts the disease progression and quality of life of AECOPD patients. Therefore, for AECOPD patients with elevated WBC levels, close monitoring of their inflammatory status and sleep patterns is essential, with early interventions to reduce the risk of insomnia and improve clinical outcomes.

Furthermore, the study found that EOS% plays a significant role in predicting insomnia in patients with AECOPD. An elevated eosinophil percentage may influence the pathophysiological processes in AECOPD patients through multiple mechanisms. First, eosinophil-mediated Th2-type inflammatory responses (such as the release of IL-5 and IL-13) may enhance systemic inflammation levels (32), which could potentially cross the blood–brain barrier and interfere with the sleep-regulating function of the HPA axis (28). Second, an increase in EOS% not only reflects an increased systemic inflammatory burden but may also exacerbate airway inflammation, leading to airway hyperreactivity and nocturnal breathing difficulties, which significantly impair sleep quality. Additionally, during the acute phase of AECOPD, patients often experience symptoms such as nighttime dyspnea and coughing, which are closely associated with excessive mucus secretion caused by eosinophils, further contributing to sleep fragmentation and reduced sleep quality (33). Therefore, in clinical practice, it is important to monitor EOS% levels in AECOPD patients with insomnia, using it as a key indicator for identifying those at high risk for insomnia. Interventions targeting systemic inflammation could effectively improve sleep quality while potentially slowing the further progression of AECOPD.

PSQI score, marital status, WBC levels, and EOS% are closely associated with insomnia in various diseases. PSQI, an important tool for assessing sleep quality, is frequently linked to insomnia symptoms and psychological issues in patients with chronic conditions such as cardiovascular diseases, diabetes, and cancer (34, 35). Marital status, particularly widowhood, often leads to heightened feelings of loneliness, emotional trauma, and increased psychological stress, which can exacerbate insomnia, especially in the elderly and those with chronic illnesses. WBC levels, as an inflammation marker, are also elevated in conditions like rheumatoid arthritis (36), potentially interfering with sleep regulation through pro-inflammatory factors. Increased EOS% is associated with nocturnal symptoms in diseases like asthma (37), which can lead to sleep disturbances and worsen insomnia through inflammation and oxidative stress.

This study has several strengths. First, it incorporates a comprehensive analysis of various factors, including clinical characteristics, laboratory tests, and treatment measures, with a specific focus on the impact of corticosteroids, quinolone antibiotics, and mechanical ventilation on insomnia. This approach makes the study more thorough and insightful. Second, we developed, for the first time, a risk prediction model for insomnia in patients with AECOPD, which can assist clinicians in assessing the likelihood of insomnia and help in formulating more comprehensive clinical treatment plans to improve COPD symptoms and outcomes. Additionally, the four predictive factors identified in our study are easily accessible in practice, making the model practical and simplifying the assessment process, thereby offering significant clinical value. Finally, to validate the model, we employed ROC curves, calibration curves, DCA, and the Hosmer-Lemeshow goodness-of-fit test, and the results demonstrated the model’s excellent performance.

This study also has some limitations. First, as a single-center study, there may be regional and population-specific limitations, which could affect the generalizability of the results. In the future, we plan to include patients from different regions to further validate the model and improve the applicability of the findings. Additionally, we aim to expand the scope of the study to include patients with AECOPD and comorbidities such as anxiety, depression, and bipolar disorder, in order to explore more potential intervention strategies. We also plan to conduct a multicenter study to enhance the generalizability and reliability of the research. Moreover, future studies will focus on strengthening patient follow-up, assisting clinicians in implementing personalized interventions, including pharmacological treatments (e.g., sleep aids), cognitive behavioral therapy for insomnia (CBT-I), breathing exercises, pulmonary rehabilitation, and lifestyle changes (such as improving sleep environment, dietary, and exercise recommendations). Regular assessments and adjustments to treatment strategies will help improve the quality of life for COPD patients.

## 5 Conclusion

This study analyzed clinical, pathological, laboratory, and treatment-related data of patients with AECOPD and developed a risk prediction model for insomnia in these patients based on four predictive factors: PSQI score, marital status (widowed), WBC levels, and EOS%. A nomogram was also created to visualize the model. The application of this model will aid in identifying high-risk individuals for insomnia among patients with AECOPD, thereby optimizing clinical treatment decisions, enabling timely interventions, improving sleep quality, and enhancing the overall quality of life for these patients.

## Data Availability

The raw data supporting the conclusions of this article will be made available by the authors, without undue reservation.
